# A Novel Variant of Treacle Ribosome Biogenesis Factor 1 (TCOF1) Gene Manifesting as Treacher Collins Syndrome

**DOI:** 10.7759/cureus.66873

**Published:** 2024-08-14

**Authors:** Tanya Tandon, Abhinav Thakral, Divya Moorthy, Kimia Satani, Kim Roger

**Affiliations:** 1 Pediatrics, Brookdale University Hospital Medical Center, Brooklyn, USA; 2 Genetics, Maimonides Medical Center, Brooklyn, USA; 3 Neonatology, Brookdale University Hospital Medical Center, Brooklyn, USA

**Keywords:** treacher collins syndrome (tcs), pathological variations, a new variant, neonatal care, pediatric genetics

## Abstract

Treacher Collins syndrome (TCS) is a rare genetic disorder. The clinical presentation of this syndrome can vary among members of the same family. The commonly associated genes with TCS are mostly inherited as autosomal dominant; however, rare autosomal recessive inheritance has been reported. We present the case of a one-day-old term male baby diagnosed with TCS wherein the genetic workup revealed a novel pathogenic variant on exon 17, which has not been reported in the literature previously. This is the first case report regarding a novel pathogenic variant within exon 17 of the Treacle Ribosome Biogenesis Factor 1 (*TCOF1*) gene causing Treacher Collins syndrome.

## Introduction

Treacher Collins syndrome (TCS) is a rare genetic disorder affecting one in 25,000 to one in 50,000 live births [1]. The presentation can vary widely even among affected members of the same family. This syndrome is primarily inherited in an autosomal dominant manner, although rare cases of autosomal recessive inheritance have been reported. Some commonly known genetic mutations associated with TCS occur in the Treacle Ribosome Biogenesis Factor 1 (*TCOF1*) gene, although other genes such as *POLR1B* and *POLR1C* have been implicated. The mutations associated with this syndrome disrupt the development of facial bones and tissues, resulting in the underdevelopment of the zygomatic complex, cheekbones, jaws, palate, and mouth, which can lead to significant complications including breathing and feeding difficulties and hearing loss [2]. As the severity and symptoms of TCS vary dramatically from remaining undiagnosed to severe life-threatening respiratory distress, it becomes imperative for healthcare workers to have a high index of suspicion when coming across these characteristic dysmorphisms.

In our case report, we present a one-day-old term male baby born to unaffected parents with advanced maternal and paternal age in an otherwise healthy family, with facial and auricular deformities needing advanced delivery room resuscitation efforts. Genetic testing done during neonatal intensive care unit (NICU) stay revealed a novel pathogenic variant in exon 17 of the *TCOF1* gene. Such a variant has not been previously reported in the literature. This report further highlights the importance of considering TCS in neonates presenting with craniofacial abnormalities and underscores the role of detailed genetic analysis in identifying a novel pathogenic variant. The identification of this new mutation in exon 17 of the *TCOF1* gene contributes to the understanding of the genetic basis of TCS and its clinical variability, providing valuable information for future research.

## Case presentation

We present the case of a one-day-old male baby born at 38 6/7 weeks of gestation via normal spontaneous vaginal delivery in a community hospital to a 42-year-old G4P1021 Haitian mother and a 49-year-old Haitian father. The Apgar scores given at birth were 9 at one minute and 9 at five minutes. A brief physical examination revealed bilateral external ear canal deformities with dysmorphic facial features. Birth demographics were appropriate for gestation with a birth weight of 3,375 g, length of 50 cm, and head circumference of 35 cm, 54%, 48%, and 65% on the Fenton curve, respectively. The neonate was successfully transported to the NICU on continuous positive airway pressure (CPAP) with positive end-expiratory pressure (PEEP) of 5% and 21% FiO2 due to mild distress at rest and was soon transitioned to room air as saturations were maintained above 95% at all times. Maternal chart review showed consistent prenatal visits and use of routine prenatal vitamins. Prenatal imaging of a detailed fetal anatomy ultrasound examination was reported remarkable for micrognathia and low-set ears with adequate amniotic fluid and weight for gestation. A follow-up sonogram for fetal facial anatomy was recommended, but the mother did not keep her appointment. Prenatal chromosomal analysis showed 46+ XY confirmed on amniocentesis. The acetylcholine esterase levels were negative, and no microarray testing results were available. Family history review was notable for two older siblings who were healthy and did not exhibit any developmental or intellectual concerns or dysmorphic features. Parents themselves had finished college and did not have any known medical conditions; they denied consanguinity or other sexual relations.

**Figure 1:**
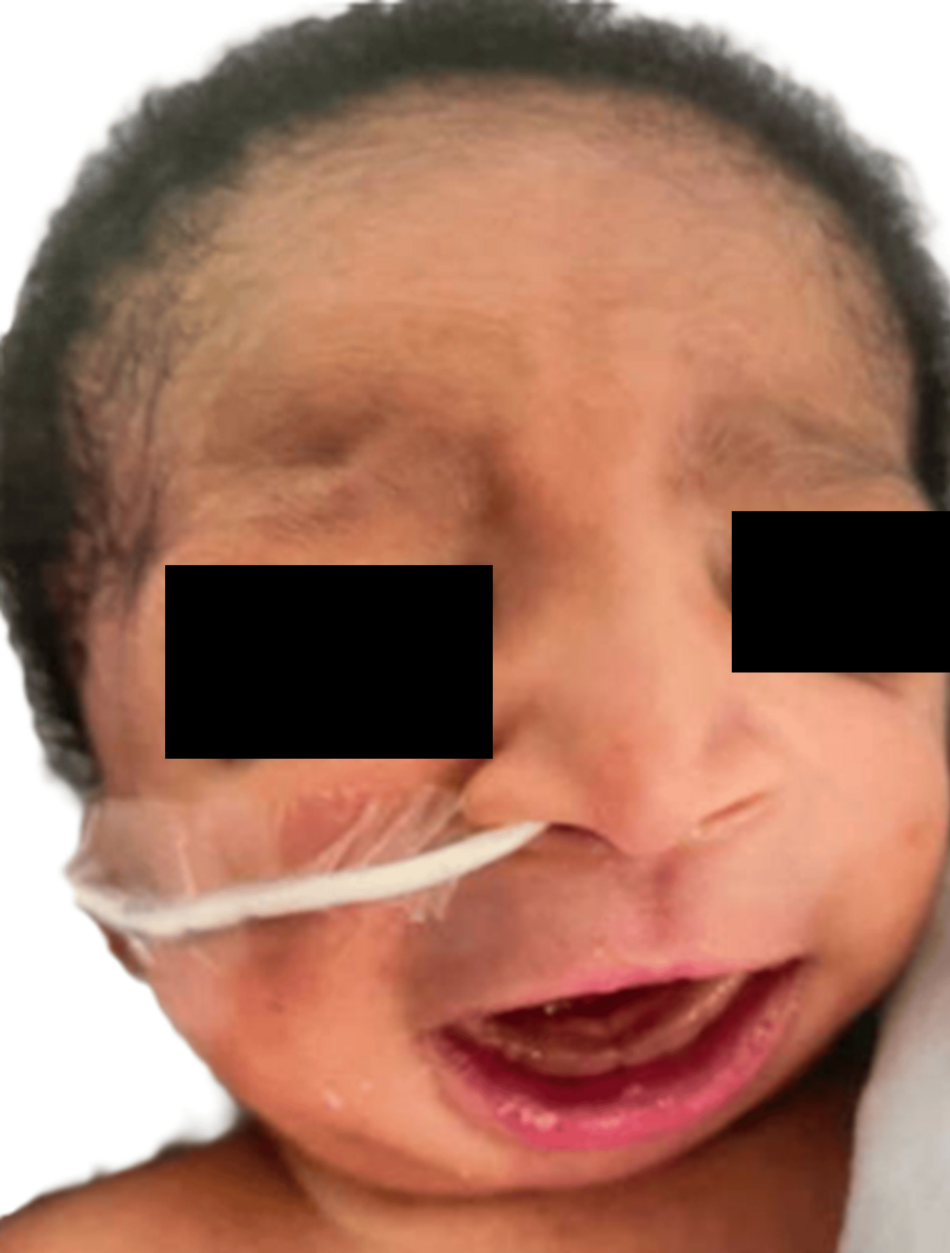
Facial dysmorphism: ocular hypertelorism and broad nasal bridge with retro-micrognathia A nasogastric tube was placed because of feeding difficulty due to micro-retrognathia.

**Figure 2:**
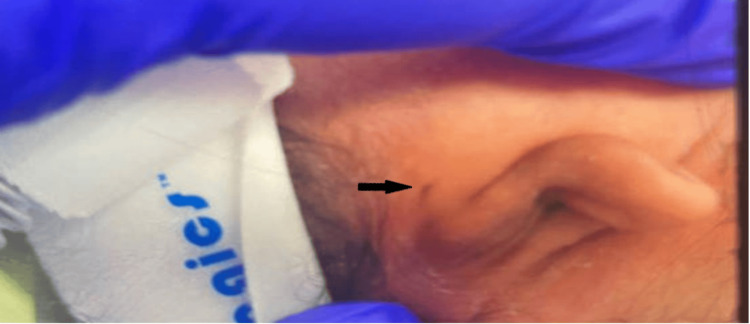
Auricular microtia with auricular pit visible above the right ear (black arrow)

**Figure 3:**
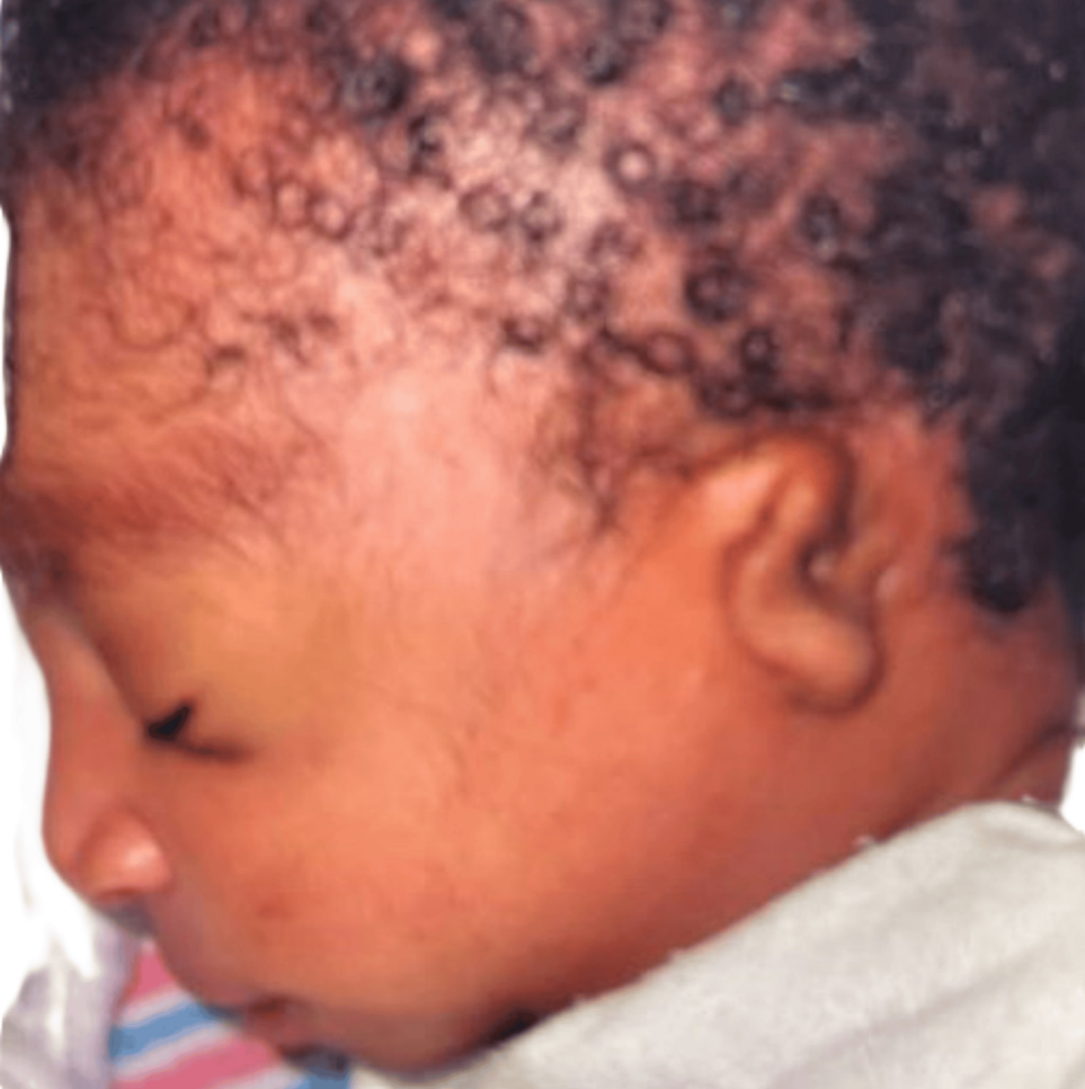
Microtia with low-set ear visible on the left side Micrognathia is also appreciated.

**Figure 4:**
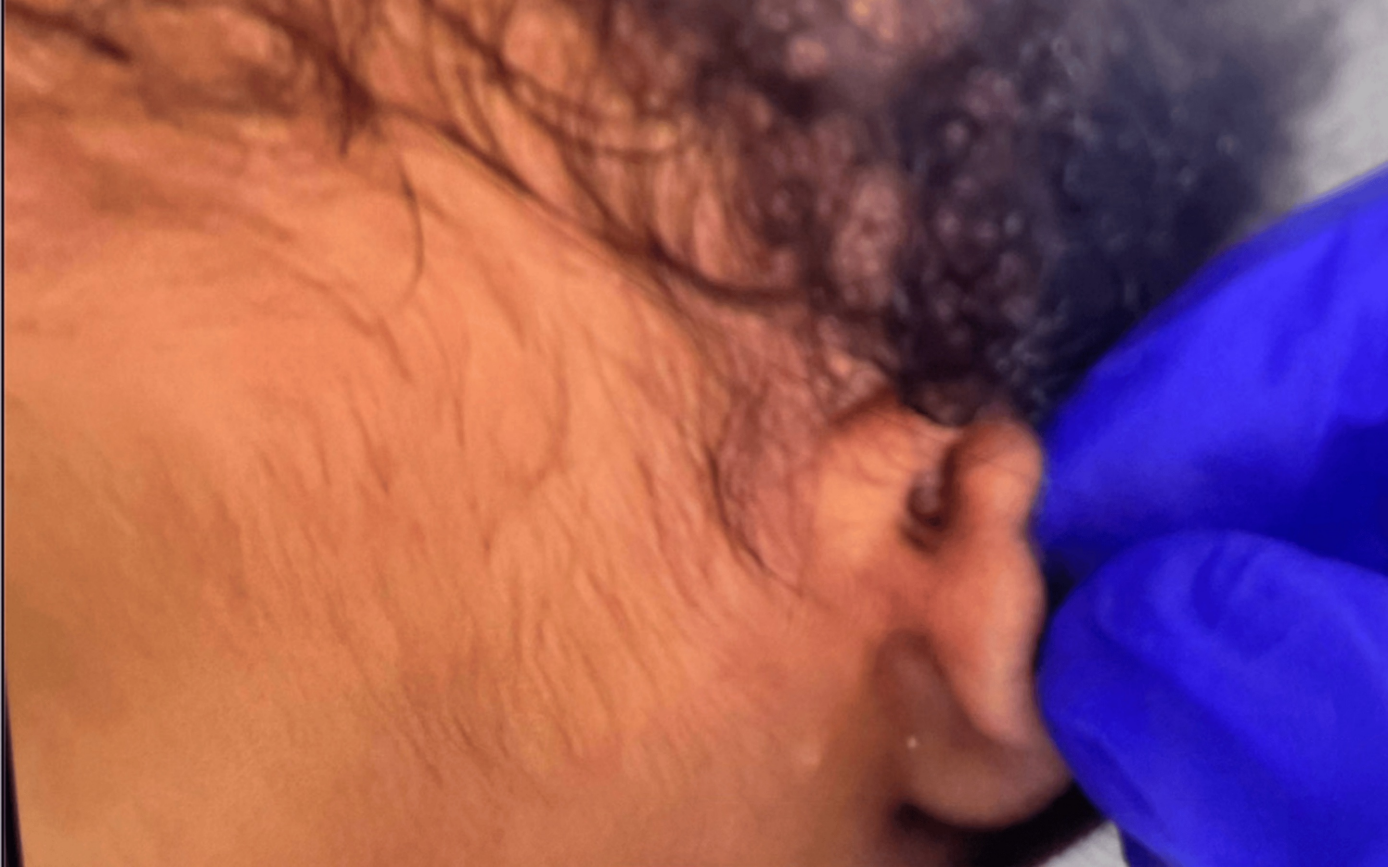
Absent external auditory canal

**Figure 5:**
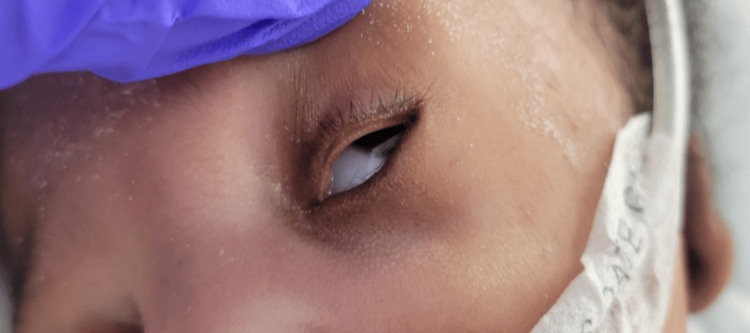
External ocular examination shows notching of the lower eyelid

A thorough physical examination in the NICU revealed an appropriately flexed posture and tone, bilateral microtia, low-set ears, absent external auditory canal opening with pits present superiorly, ocular hypertelorism, and broad nasal bridge with retro-micrognathia (Figures 1-4). The oral examination showed an intact palate on palpation with a good suck reflex. The abdominal examination showed wide-spaced nipples and bilaterally descended testes, with a symmetrical Moro reflex. No organomegaly was palpable. The limbs examination was normal with no evidence of pre- or post-axial polydactyly. At rest, the newborn was noted to have grunting noises owing to the micro-retrognathia; however, no dips in saturation were recorded. Feeding was continued through the nasogastric tube. Bedside imaging studies with abdominal and head sonograms were reported unremarkable with no identifiable renal or brain abnormalities. Plain radiograph films showed the presence of a thymic shadow with no spinal deformities. Comprehensive metabolic panel (CMP) studies reported no electrolyte imbalances; hence, CATCH-22 syndrome seemed unlikely. Multispecialty team evaluation reported an echocardiogram with trivial mitral and tricuspid valve regurgitation and small atrial septal defect (ASD). The ophthalmology examination was unremarkable on the dilated fundus examination, but the anterior segment examination in both eyes showed atresia of lower lid puncta (Figure 5). Hearing test done using auditory brainstem response failed bilaterally.

On an initial review of history and physical examination, the team considered the possibility of Treacher Collins syndrome as the primary differential due to characteristic dysmorphic facial features. Other differentials considered on the list included​ Pierre Robin sequence, Goldenhar syndrome, CHARGE syndrome (coloboma, heart defects, atresia choanae, growth retardation, genital abnormalities, and ear abnormalities),​ lacrimo-auriculo-dento-digital (LADD) syndrome,​ isolated microtia,​ oculo-vertebral syndrome​, oculo-oral syndrome,​ and DiGeorge syndrome. In coordination with our genetics team, testing with single nucleotide polymorphism (SNP) microarray and fluorescence in situ hybridization (FISH) for DiGeorge and Treacher Collins syndrome (TCS) panels including most commonly associated genes were sent, which included *SF3B4*, *TCOF1*, *POLR1D*, *POLR1C*, *HODH*, and *EFTUD2*. The TCS panel reported an autosomal dominant mutation on the *TCOF1* gene, pointing to the heterozygous inheritance with a new pathogenic variant on exon 17, namely, c.2689A>T (p.Arg897*). A multidisciplinary family meeting was conducted, and given the mode of inheritance reported in the background of a negative family history, the likely cause of the mutation in our patient was considered to be de novo or germline mutation in either parent or rarely incomplete penetrance with almost no dysmorphic features in the parent who carries the gene mutation. Parental genetic counseling was offered; however, the parents refused any further genetic testing or counseling.

## Discussion

Treacher Collins syndrome (TCS) is a rare genetic disorder characterized by distinctive abnormalities of the head and face, causing underdevelopment of the zygomatic complex, cheekbones, jaws, palate, and mouth, which can lead to breathing and feeding difficulties and anomalies of external and middle ear structures, which may result in hearing loss [1]. Prevalence is estimated to range between one in 25,000 and one in 50,000 live births [2]. The symptoms and severity of TCS can vary drastically dramatically from one person to another; thus, both incomplete penetrance and variable expressivity are possible, even among members of the same family. Some individuals may be so mildly affected that they can remain undiagnosed, while others may have significant abnormalities and the potential for life-threatening respiratory complications. TCS shows genetic heterogeneity as the syndrome can be caused by mutations of different genes including the *TCOF1*, *POLR1B*, *POLR1C*, or *POLR1D* genes. For our patient, the TCS panel test reported autosomal dominant *TCOF1* gene mutation heterozygous inheritance with a pathogenic variant on exon 17, namely, c.2689A>T (p.Arg897*). The Treacle Ribosome Biogenesis Factor 1 (*TCOF1*) gene is located in the chromosomal region 5q32-q33.1. The gene encodes a nucleolar protein that serves several important roles, including regulation of RNA polymerase 1. The protein is also involved in neural crest cell specification.

The mode of inheritance for Treacher Collins syndrome (TCS) associated with *TCOF1* mutations is autosomal dominant, although very rare cases of autosomal recessive mutations have been observed [1]. The spectrum of mutations within the *TCOF1* gene that causes TCS is very heterogeneous. The majority of the mutations are insertions and deletions that result in premature termination codons [3]. About 55%-61% of probands have the disorder as the result of a de novo *TCOF1* pathogenic variant [4]. An extensive literature review of known mutations for exon 17 in the *TCOF1* gene revealed no previous case reports for this specific mutation in *TCOF1 *exon 17, c.2689A>T (p.Arg897*), which is a novel pathogenic variant to cause Treacher Collins syndrome in our neonate. The c.2689A>T (p.Arg897*) sequence change creates a premature translational stop signal (p.Arg897*) in the *TCOF1* gene. Hence, it is expected to result in an absent or disrupted protein product, presumably leading to altered regulation of the RNA polymerase 1 and neural crest cell specification. Hence, this loss-of-function variant in *TCOF1* is deemed pathogenic [5]. This variant has not been reported in any population database as assessed on the Genome Aggregation Database (gnomAD) (National Institutes of Health (NIH)). This variant has not been reported in the literature in individuals affected with *TCOF1*-related conditions. This is the first nonsense variant reported for exon 17 of the *TCOF1* gene as per a thorough ClinVar search.

## Conclusions

In conclusion, our case of the one-day-old term male baby born at 38 6/7 weeks of gestation to a 42-year-old Haitian mother and a 49-year-old Haitian father, with a diagnosis of Treacher Collins syndrome (TCS) confirmed through the identification of a novel pathogenic variant, c.2689A>T (p.Arg897*), in the *TCOF1* gene brings forth multiple aspects of care into consideration. This novel mutation, which had not been previously documented in the literature, underscores the genetic diversity and complexity inherent to TCS and highlights the crucial role of genetic testing in diagnosing rare disorders with variable expressivity. This novel mutation located on exon 17 of the *TCOF1* gene created a premature stop codon that disrupted protein production and function, leading to the characteristic features of TCS, such as bilateral microtia, low-set ears, and absent external auditory canal openings.

The family's decision to decline further genetic counseling or testing highlights the profound challenges and emotional burden associated with managing a child with a genetic disorder. The difficulty in coping with the diagnosis, concerns about potential implications for future pregnancies, a desire to focus on immediate care and support for the child, and the financial burden associated with it are but a few challenges. It therefore becomes critical for a healthcare team to be reminded of the impact a genetic disorder can have beyond the immediate stabilization and care during the hospital stay. This situation underscores the importance of providing compassionate and comprehensive support to families facing genetic disorders, ensuring families have access to the resources and counseling needed to make complex informed decisions and manage the quality of life for their child in a shared decision-making process. The multidisciplinary approach highlighted in managing our case, encompassing geneticists, neonatologists, and other specialists, demonstrates the importance of collaborative care. This case study clearly shows the need for continued research into genetic disorders such as Treacher Collins syndrome to better understand the underlying mechanisms, improve diagnostic techniques, and develop targeted interventions. The discovery of this novel mutation serves as a critical reminder of the intricate balance a genetic disorder can have on family dynamics and the role of a comprehensive healthcare team.
